# “*I take it and give it to my partners who will give it to their partners”:* Secondary distribution of HIV self-tests by key populations in Côte d’Ivoire, Mali, and Senegal

**DOI:** 10.1186/s12879-023-08319-4

**Published:** 2023-05-24

**Authors:** Odette Ky-Zerbo, Alice Desclaux, Sokhna Boye, Mathieu Maheu-Giroux, Nicolas Rouveau, Anthony Vautier, Cheick Sidi Camara, Brou Alexis Kouadio, Souleymane Sow, Clémence Doumenc-Aidara, Papa Alioune Gueye, Olivier Geoffroy, Odé Kanku Kamemba, Eboi Ehui, Cheick Tidiane Ndour, Abdelaye Keita, Joseph Larmarange, for the ATLAS team

**Affiliations:** 1 TransVIHMI, Université de Montpellier, IRD, INSERM, Montpellier, France; 2 Ceped, Université Paris Cité, IRD, Inserm, Paris, France; 3 Department of Epidemiology and Biostatistics, School of Population and Global Health, McGill University, Montréal, QC H3A 1G1 Canada; 4 SOLTHIS, Dakar, Sénégal; 5 Institut Malien de Rechercher en Sciences Sociales Et de Santé (IMRSS), Bamako, Mali; 6 Institut d’ethnosociologie (IES), Université Félix Houphouët Boigny de Cocody, Abidjan, Côte d’Ivoire; 7 Centre Régional de Recherche et de Formation à la Prise en Charge Clinique de Fann (CRCF), Dakar, Sénégal; 8 SOLTHIS, Abidjan, Côte d’Ivoire; 9 SOLTHIS, Bamako, Mali; 10 Programme National de Lutte contre le Sida (PNLS), Abidjan, Côte d’Ivoire; 11 Division de Lutte contre le Sida et les IST, Ministère de la Santé et de l’Action Sociale, Institut d’Hygiène Sociale, Dakar, Sénégal; 12 Institut National de Santé Publique (INSP), Bamako, Mali

**Keywords:** HIVST, Secondary distribution, Key population, ATLAS, West and Central Africa

## Abstract

**Introduction:**

HIV epidemics in Western and Central Africa (WCA) remain concentrated among key populations, who are often unaware of their status. HIV self-testing (HIVST) and its secondary distribution among key populations, and their partners and relatives, could reduce gaps in diagnosis coverage.

We aimed to document and understand secondary HIVST distribution practices by men who have sex with men (MSM), female sex workers (FSW), people who use drugs (PWUD); and the use of HIVST by their networks in Côte d’Ivoire, Mali, and Senegal.

**Methods:**

A qualitative study was conducted in 2021 involving (a) face-to-face interviews with MSM, FSW, and PWUD who received HIVST kits from peer educators (primary users) and (b) telephone interviews with people who received kits from primary contacts (secondary users). These individual interviews were audio-recorded, transcribed, and coded using Dedoose software. Thematic analysis was performed.

**Results:**

A total of 89 participants, including 65 primary users and 24 secondary users were interviewed. Results showed that HIVST were effectively redistributed through peers and key populations networks. The main reported motivations for HIVST distribution included allowing others to access testing and protecting oneself by verifying the status of partners/clients. The main barrier to distribution was the fear of sexual partners’ reactions. Findings suggest that members of key populations raised awareness of HIVST and referred those in need of HIVST to peer educators. One FSW reported physical abuse.

Secondary users generally completed HIVST within two days of receiving the kit. The test was used half the times in the physical presence of another person, partly for psychological support need. Users who reported a reactive test sought confirmatory testing and were linked to care. Some participants mentioned difficulties in collecting the biological sample (2 participants) and interpreting the result (4 participants).

**Conclusion:**

The redistribution of HIVST was common among key populations, with minor negative attitudes. Users encountered few difficulties using the kits. Reactive test cases were generally confirmed. These secondary distribution practices support the deployment of HIVST to key populations, their partners, and other relatives. In similar WCA countries, members of key populations can assist in the distribution of HIVST, contributing to closing HIV diagnosis gaps.

## Background

West and Central Africa (WCA) has the third highest burden of people living with HIV in the world. In this region, key populations and their sexual partners accounted for 74% of new HIV infections in 2021 [1]. Countries have different profiles, as HIV prevalence in Côte d’Ivoire, Mali, and Senegal was respectively 1.9%; 0.8% and 0.3% among adults in 2021 [2]. Men who have sex with men (MSM) are more likely to be living with HIV (8% in Côte d’Ivoire, 13% in Mali and 28% in Senegal) than Female sex workers (5% in Côte d’Ivoire and Senegal; 9% in Mali) and People who use drugs (PWUD) (8% in Côte d’Ivoire and 4% in Senegal) [3]. Less HIV positive people know their HIV status compared to Eastern and Southern African countries: 91% of adults living with HIV are aware of their status in Eastern and Southern Africa versus 84% in WCA [1]. Since 2016, World Health Organisation (WHO) recommands HIVST as a delivery model, which can help closing HIV testing gap [4]. HIVST is a process in which the user takes a sample (oral fluid or blood), performs the HIV test, and then interprets the result by himself, often in a private setting.

HIVST can be provided through a variety of strategies. Primary HIVST distribution refers to the direct provision of HIVST to users. Social-network strategies to deliver HIVST can be developed through secondary distribution, where primary contacts are invited to redistribute HIVST kits to their peers, partners, clients, and relatives. HIVST allows the user to perform the test with full confidentiality [5, 6]. It also reduces geographic and financial barriers to testing services when kits are distributed to users through outreach strategies. HIVST can reduce the stigma experienced by some users accessing “traditional” HIV testing modalities [4–6]. Key populations such as MSM, FSW and PWUD often experience stigma and discrimination, self-isolation, and punitive laws that act as barriers to HIV testing [7].

The HIVST strategy has been more commonly employed in Eastern and Southern Africa, than in WCA. Studies have found that HIVST is accepted by MSM and FSW, who are able to use and interpret the oral HIVST results correctly [8–12]. It allows to reach more MSM and FSW first-time testers [13] and to provide care services [10]. Primary HIVST distribution may be less likely to reach key populations that do not use existing services, in particular those who do not self-identify as FSW, MSM, or PWUD, and for which secondary distribution could be useful. In Zambia and Lesotho, the secondary distribution of HIVST through a door-to-door strategy has been effective. For example, men who are often not at home when testing teams visit can use test kits procured for them by their wives when they want [14, 15]. In Côte d’Ivoire, Mali, and Senegal, analyses conducted among MSM, FSW, and PWUD have shown that secondary distribution is a resilient strategy that can be used in times of crisis, such as the COVID-19 pandemic, for the continuous provision of HIV testing services to key populations [16]. Other authors concluded that key populations in WCA, where they face a double stigma (HIV-related stigma combined to sexual behaviour and practices stigma) that limits demand for testing services [17, 18], have favourable attitudes about HIVST kits distribution to their partners and other relatives.

However, in WCA, secondary distribution practices have not been studied in depth. Most studies so far have used quantitative approaches and have lacked the qualitative nuances required to understand particular contexts where HIV is less prevalent and stigma levels are higher. Also, in WCA, qualitative studies examined attitudes to and perceptions on HIVST secondary distribution, and not on their actual practices. Specifically, there is little data available on distribution of HIVST within key populations’ networks with otherwise difficult access to facility-based HIV testing. For example, what are the motivations and barriers to secondary distribution? What are the strategies used by key populations for distribution to partners, peers, clients and other relatives? What are the HIVST practices after receiving the kit? Some studies examining perception related to the introduction of HIVST among stakeholders in Africa generally casts doubt on the effective use of the distributed kits, the capacity of key populations to use HIVST and correctly interpret the results [5, 19–22], and the ability of those populations to access care services [21–23].

Using qualitative methods, the objective of this paper is to analyse secondary HIVST distribution practices by MSM, FSW and PWUD, as well as the use of HIVST by their partners, peers and social contacts who received the kits through secondary distribution in Côte d’Ivoire, Mali, and Senegal.

## Materials and methods

### Study context

This analysis was undertaken within the ATLAS project which the research protocol is published elsewhere [24]. Funded by Unitaid, the ATLAS programme aims to promote and deploy HIVST in three West African countries (Côte d’Ivoire, Mali, Senegal). The OraQuick HIV Self-Test® was selected by countries and used in this program. Considering the concentrated nature of HIV epidemics in West Africa, the ATLAS programme prioritized key populations and their sexual partners, peers, and clients, as well as people with sexually transmitted infections, their partners, and partners of PLHIV. The ATLAS program distributed HIVST to FSW and MSM as key populations in the three countries, and to PWUD only in Côte d’Ivoire and Senegal. The distribution approach combined fixed and outreach strategies and employed both primary and secondary distribution of HIVST kits. Between July 2019 and January 2022, ATLAS distributed around 400 000 kits, including 91% to key populations (254 293 FSW, 94 775 MSM, 13 096 PWUD).

ATLAS delivery of HIVST to members of key populations starts at fixed or outreach sites. HIVST kits are provided by trained health providers and mostly by peer educators (85% of distribution). The peer educators explain to members of key populations who wish to use HIVST how to use the kits, how to interpret the results, and the requirement to seek confirmatory testing if the result is reactive. Peer educators further refer users to the materials included in the HIVST kit: the manufacturer’s instructions and a complementary explanatory brochure, produced by the project. Peer educators have access to videos in French and various national languages that contain the same information, which they can share with other persons as needed. A free hotline number is provided to support HIVST users and/or for referral in case of reactive tests. Users also have the option of being assisted in-person by peer educators in performing HIVST; alternatively, they can do so on their own at a time and place of their choice. There is no obligation to share the results with the peer educators who facilitate HIVST. In this article, users at this first level are referred as “*primary users”*.

Peer educators provide kits to primary users that can be shared with members of their social network (partners, clients, hidden peers, relatives), after assessing their needs and providing brief training concerning how to distribute HIVST. It was recommended that a maximum of 1 to 5 kits be distributed per primary user. The exact number of kits was determined by the peer educators who identified with the primary users the people eligible for HIVST in his/her social network. In this article, people to whom primary users provide HIVST kits are referred to as “*secondary users*”. In turn, these users may provide kits to others, referred to as “*tertiary users*”. Secondary or tertiary users may not be members of key populations. More than 30% of the kits were distributed by primary contacts to their peers, partners, and clients.

Further details on the ATLAS project can be found in the research protocol [25]. Further, project’s results have been published on stakeholders’ attitudes and perceptions on HIVST in West Africa [21], the relevance of HIVST secondary distribution to resilient testing services [16], challenges faced by HIVST secondary distribution in contexts of low disclosure of HIV test result [26], FSW’s willingness to distribute HIVST kits [27], and the impact of HIVST in Côte d’Ivoire [28]. Cost and scale-up costs of integrating HIVST in community-led programs for key population were also published [29].

### Data collection

This article is based on data taken from two qualitative studies and focuses on secondary HIVST distribution among MSM, FSW, PWUD, and their respective social networks (PWUD were not interviewed in Mali). The studies conducted consisted of (i) individual face-to-face interviews to analyse the use and secondary distribution of HIVST by MSM, FSW, and PWUD who received the kits from peer educators and (ii) phone interviews to analyse the practices involved in HIVST kits distribution from the perspective of “*secondary recipients*” as well as the ability of those recipients to perform self-testing and to seek confirmation and care services in case of a reactive result.

**Fig. 1:**
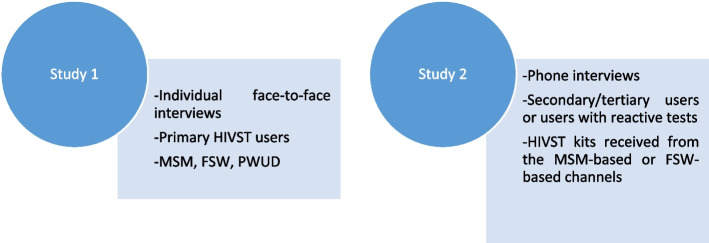
Study design: Qualitative data collection on HIV self-test (HIVST) secondary distribution and utilization among key populations and their network in Côte d’Ivoire, Mali and Senegal, 2021

The face-to-face interviews with primary users were conducted between January-April of 2021. The phone interviews with secondary or tertiary users were conducted between June–August of that same year. The interviews lasted between 40 min (phone interviews) to one hour (face-to-face interviews). Before the beginning of the interviews, basic data concerning the sociodemographic profile of participants were collected. The interviews were conducted in French and/or in national languages (Bambara in Mali, Wolof in Senegal) according to the preferences of the participants. All participants in Côte d’Ivoire preferred to be interviewed in French. The same researchers conducted the face-to-face and phone interviews (KBA in Côte d’Ivoire, CSC and KZO in Mali, SS in Senegal). Interviewers had a minimum of a master’s degree in social sciences or public health and they were originally from the same country and racial group as interviewees; they were experienced in data collection with vulnerable key populations and were familiar with the groups and organizations or clinics where the interviews were conducted. The study design is presented in Fig. 1.

#### Individual face-to-face interviews

Interviews were conducted in urban and semiurban settings in which HIVST were distributed using both fixed and outreach strategies: Abidjan and San Pedro in Côte d’Ivoire, Bamako and Kati in Mali, and Dakar and Mbour in Senegal. Peer educators invited MSM, FSW, and PWUD to whom they had provided HIVST kits to take part in the study, regardless of the period of the kits’ receipt, the distribution to their network or the test’s result. A semistructured interview guide was used during the interviews, which addressed five topics: i) the motivations and circumstances for HIVST use, ii) first experiences with HIVST, iii) social experiences of HIVST use (i.e., distribution to and use by family members and related consequences), iv) confirmation and management pathways, and v) participants’ suggestions.

#### The phone interviews

Phone interviews were conducted with two types of users. Those who i) had received their HIVST kits from MSM or FSW who were not peer educators and those who ii) declared that their HIVST result was reactive. Participants were recruited from a related quantitative study (phone survey) conducted between March-June 2021. For this phone survey, a flyer was distributed with the HIVST kits that invited individuals to call an anonymous toll-free number to participate in a short survey. A unique participation number on each flyer identified the original distribution channel of the HIVST kits (i.e., FSW, MSM, PWUD). From this phone surveys, participants were further recruited for this qualitative study. They were selected based on the following criteria: i) having completed the quantitative survey; ii) having consented during the quantitative survey to be contacted again for a complementary qualitative component; iii) having provided a telephone number to be called back; and iv) having been recruited via the FSW or MSM channels and/or having declared that their HIVST result was reactive. The dates and times of the interviews were determined in advance in collaboration with participants. The phone interview guide focused on 5 topics: i) the modalities of access to HIVST via secondary distribution, ii) first experiences of HIVST, iii) participants’ opinions of HIVST, iv) experiences of tertiary HIVST distribution, and V) the healthcare trajectories of people who declared a reactive test result.

### Data management and analysis

The interviews were audio-recorded with the consent of the participants. During transcription, the interviews were translated to French when necessary by one of the researchers from each country. The study’s field coordinator (the first author) proofread the transcripts. The content of the edited versions was pseudonymized by removing the names of people and places mentioned by the participants. From the interview guide topics and the content of the interviews, codes and subcodes were first identified by the field coordinator. A coding base was created using Dedoose® software. A pretest was conducted by two researchers (KZO and CSC) and an independent consultant (Barro Saran). They first coded the same interviews separately, compared results, and discussed the similarities and differences to reach a consensus. The coding tree was corrected before the data was coded by the same researchers. The coding report was then exported into Word by code/subcode, and by key population profile. Data memos were written first for each code in turn, and a thematic analysis was performed. The themes were deductively informed from the interview guide, while additional themes were identified from interview content. Emerging themes were compared between FSW, MSM and PWUD.

For this article, we analysed the themes that emerged from data i) the social experience of HIVST distribution among primary users who were interviewed face-to-face and ii) the modalities of access to self-testing, the experiences of first-time HIVST users, and the tertiary HIVST distribution experiences of secondary users who were interviewed by phone. The care itineraries were analysed for primary and secondary users who declared a reactive test.

### Ethical considerations

The research protocol and the various tools used were approved by the ethics committees of the study countries and the WHO: the WHO Ethical Research Committee (07 August 2019, reference: ERC 0,003,181); the National Ethics Committee for Life Sciences and Health of Côte d’Ivoire (28 May 2019, reference: 049–19/MSHP/CNESVS-kp); the Ethics Committee of the Faculty of Medicine and Pharmacy of the University of Bamako, Mali (August 14, 2019, reference: 2019/88/CE/FMPOS); and the National Ethics Committee for Health Research of Senegal (July 26, 2019, protocol SEN19/32). Before each interview, an information sheet was read to participants and translated into a national language when necessary. All participants in the face-to-face interviews signed a consent form regarding their participation in the study and the recording of the interviews, while participants in the telephone study provided verbal consent. The completed forms and audio recordings of the interviews will be destroyed at the end of the project. The face-to-face interviews were conducted in private spaces located within NGO structures. No names were recorded, as each participant was assigned a participation number. The phone interviews were conducted at a time chosen by the participant, which we assume corresponded to a time when they could be surrounded by the secure conditions required for participation in the study. When participants were contacted again regarding the qualitative component, verbal consent was once again collected. For reasons of confidentiality, information concerning the geographic location of participants was not collected during the interviews.

## Results

### Description of participants

The two studies involved a total of 89 people: 65 participants (73%) in the face-to-face interviews and 24 participants (27%) in the phone interviews.

#### Face-to-face interviews

Individual face-to-face interviews were conducted with 21 MSM (32%), 24 FSW (37%), and 20 PWUD (31%). The sociodemographic characteristics of the participants are presented in Table 1. Three PWUD were female. PWUD appeared to be older than MSM and FSW, with an average age of 44 years for PWUD versus averages of 25 and 27 years for MSM and FSW, respectively. MSM and FSW were more often single than PWUD (21/22 MSM, 17/24 FSW, 12/20 PWUD). MSM reported higher levels of education than FSW and PWUD. Indeed, 19/21 MSM, 14/24 FSW, and 10/20 PWUD had received at least a secondary education. Approximately half of the participants had used HIVST more than once in their lifetime (36/65). The frequency of HIVST use (more than once in their lifetime) was higher among MSM (14/20) and FSW (15/24) than among PWUD (7/20). Two participants declared HIVST-reactive results (1 MSM and 1 FSW).

**Table 1:** Sociodemographic characteristics of participants in the individual face-to-face interviews by country and key populations

Sociodemographic characteristics	Côte d’Ivoire (*n* = 26)	Mali (*n* = 17)	Senegal (*n* = 22)	Total (*n* = 65)
Men who have sex with men (*n* = 21)				
Age				
18–24	5	3	3	11
25 and older	2	5	3	10
Education				
Primary	1	1	0	2
Secondary	3	5	2	10
University	3	2	4	9
Marital status				
Single	7	7	6	20
Married	0	1	0	1
HIVST utilization				
1	3	1	3	7
2 or more	4	7	3	14
HIVST Result				
Reactive	0	1	0	1
Negative	7	6	0	20
Female sex workers (*n* = 24)				
Age				
18–24	4	2	0	6
25 and older	4	7	7	18
Education				
Did not attend school	0	1	2	3
Primary	3	3	1	7
Secondary	5	4	3	12
University	0	1	1	2
Marital status				
Single	8	6	3	17
Married	0	2	0	2
Widowed/separated	0	1	4	5
HIVST utilization				
1	4	2	3	9
2 or more	4	7	4	15
HIVST Result				
Reactive	0	1	0	1
Negative	8	8	0	23
People who use drugs (*n* = 20)				
Age				
25 and older	11		9	20
Gender				
Male	10		7	17
Female	1		2	3
Education				
Did not attend school	2		0	2
Primary	4		3	7
Secondary	2		4	6
University	2		2	4
Koranic school	1		0	1
Marital status				
Single	11		1	12
Married	0		6	6
Widowed/separated	0		2	2
HIVST utilization				
1	6		7	13
2 or more	5		2	7
HIVST Result				
Reactive	0		0	0
Negative	11		9	20

#### Phone interviews

Of the 24 participants in the phone study, 16 received HIVST kits through the MSM-based channel and 8 through the FSW-based (Table 2). The majority of participants were male: 13/16 from the MSM-based channel and all ( 8/8) from the FSW-based channel. Participants who had received HIVST through the MSM-based channel had a mean age of 25 years versus a mean age of 32 years for participants who had received HIVST through the FSW-based channel. Only 2 participants from the MSM-based channel had not attended school, while all participants from the FSW-based channel had. One-third of participants had used HIVST more than once in their lifetime (7/24). Six participants had reported a reactive test (4 from the MSM-based channel and 2 from the FSW-based channel).

**Table 2:** Sociodemographic characteristics of participants in the individual phone interviews by country and secondary distribution channels

Sociodemographic characteristics	Côte d’Ivoire (*n* = 10)	Mali (*n *= 9)	Senegal (*n* = 5)	Total (*n* = 24)
Users, men who have sex with men based channel (*n *= 16)				
Age				
18–24	2	2	1	5
25 and older	3	5	3	11
Sex				
Female	1	2	0	3
Male	4	5	4	13
Education				
Did not attend school	0	2	0	2
Primary	0	0	0	0
Secondary	3	4	4	11
University	2	1	0	3
HIVST utilization				
1	2	5	4	11
2 or more	3	2	0	5
HIVST result				
Reactive	2	2	0	4
Negative	3	5	4	12
Users, female sex workers based channel (*n* = 8)				
Age				
18–24	1	1	0	2
25 and older	4	1	1	6
Sex				
Female	0	0	0	0
Male	5	2	1	8
Education				
Primary	1	1	0	2
Secondary	3	1	1	5
University	1	0	0	1
HIVST utilization				
1	4	1	1	6
2 or more	1	1	0	2
HIVST result				
Reactive	1	1	0	2
Negative	4	1	1	6

### Secondary and tertiary HIVST distribution

The majority of participants in the face-to-face interviews (primary users) reported having distributed at least one HIVST kit to a third party (45/56). Secondary distribution was often to partners (40/45) and peers (20/45). FSW reported distribution to their clients (6/24). Secondary distribution was also provided to others, such as friends, peers, and coworkers, who may not be part of the key population communities. Unlike MSM and PWUD, FSW also distributed HIVST kits to family members (Table 3).

**Table 3:** Practices of secondary HIVST distribution by primary users and profiles of the beneficiaries of secondary distribution by primary user channel (men who have sex with men, female sex workers, or people who use drugs) and country (Côte d’Ivoire, Mali or Senegal)

	MSM (*n* = 21)			FSW (*n* = 24)			PWUD (*n* = 20)			Total
	RCI	ML	SN	RCI	ML	SN	RCI	ML	SN	
Participants	7	8	6	8	9	7	11		9	65
Secondary distribution practices										
Received HIVST kits for secondary distribution	6	8	4	8	9	7	9		5	56
Distributed at least one kit	5	7	3	6	8	4	9		3	45
Relationship between primary users and recipients										
Partners	2	2	2	3	4	2	7		4	26
Peers	3	4	1	2	5	0	3		2	20
Female sex workers’ Clients				2	2	2				6
Family members	0	0	0	2	3	1	0		0	6
Other relatives	1	3	0	1	1	0	2		0	8

Distribution continues beyond the secondary users to whom HIVST kits were initially given according to a strategy adopted by some participants.

*Because he said [to give it to] my partners and their partners, because as it is, in the community, I may know someone who also knows someone, and so the distribution is done. So, I take it and I give it to my partners who will give it to their partners, and so on.* Male, 28 years old, phone interview, MSM-based channel, Mali.

One-third of secondary users reported tertiary distribution experiences (8/24), reflecting the penetration of HIVST among certain networks of key populations.

### Motivations for secondary HIVST distribution

Five main interrelated motivations for secondary HIVST distribution emerged from the interviews. Motivations for secondary HIVST distribution by members of key populations appeared to vary according to their profile and that of the secondary user.

First, with respect to participants’ main partners, the motivations reflect a certain degree of altruism, namely, through a feeling of moral obligation to protect partners by ensuring that they know their HIV status and are able to access care. This desire seemed to motivate FSW and PWUD in particular.

*You can’t do these things [have multiple sexual partners] and be in a relationship with someone without offering it to them [HIVST].* Female, 45 years old, face-to-face interview, FSW, Senegal*.*

Second, this apparent altruism appears motivated by a desire to protect themselves because condoms are rarely used with these main partners or with partners with whom participants have sex regularly. With these partners, reasons related to the need to know one’s status as a precondition of unprotected sex were also reported by MSM.

*I don’t know his status. I don’t want him to give me the virus, so I asked him to get tested before we had sex. Male,* 24 years old, face-to-face interview, MSM, Côte d’Ivoire.

Third, secondary distribution to FSW’s casual clients seems to be more closely targeted at those who refuse condoms before sex than at clients who practise safe sex. Such distribution is also targeted at regular partners with whom FSW have unprotected sex, as reported by an FSW in Mali.

*In the maquis [places of socializing such as restaurants, bars where alcohol is usually served], everyone uses condoms there. But if I go to the other client’s house, because he’s in an apartment, he doesn’t use a condom… Every time he needs me, he does the test; and if he needs an HIVST kit too [for other users], he calls me to tell me.* Female, 27 years old, face-to-face interview, FSW, Mali.

The fourth reason is simple. HIVST could serve as a means of verifying the alleged negative HIV status of a partner or client.

*Yeah, it’s three people, they don’t like to hear about that at all [using a condom]; it’s easy to say, “Hey, I did my HIV test last week”, or “I did it the day before yesterday”. So, every time they say that, and finally they get mad at me, because I say, “If you did it, show me the result”*. Male, 21 years old, face-to-face interview, MSM, Mali

Finally, secondary distribution to peers seems “natural” because primary users are convinced that all of them are exposed to the same degree of risk. Among peers, distribution is mostly to those who are reluctant to be tested or who are assumed to be at greater risk.

*Many were afraid of the needle, and I preferred to give this to someone so that they too would know if they were sick or not, so that I too would take precautions, so that I would not be infected with this disease too [during the use of shared injection materials]. That’s it, since we’re always together. So, I wanted to take precautions too.* Male, 44 years old, face-to-face interview, PWUD, Côte d’Ivoire*.*

### Barriers to secondary HIVST distribution

Between the delivery of HIVST to primary users by peer educators to its use by secondary users, some attrition occurs. However, every key population member who has used HIVST contributed to its promotion to some degree. In situations in which members of key populations perceive that they might face difficulties when offering the kits to partners or peers, they generally opted to take another approach, namely, to raise awareness among those around them and to refer anyone wishing to use HIVST to distribution sites or to peer educators. This strategy is also used when members of key populations did not have kits to distribute. In summary, challenges can arise at two levels: the peer educator level and the primary user level.

#### Peer educator-level challenges to secondary HIVST distribution

To ensure secondary distribution, kits must be available. Of the 65 primary users in the face-to-face interviews, seven said they did not have kits for secondary distribution: two MSM and five PWUD. Two primary users (1 MSM and 1 PWUD) reported refusing to take the kits for secondary distribution in anticipation of possible negative reactions from secondary users.

Among the 56 other members of key populations who received kits to distribute within their social network, 11 did not complete the distribution. Lack of communication between peer educators and primary users limited secondary distribution by some users, who said they did not understand that the additional kits were intended for members of their social network and therefore kept them for their own use.

*One thing that is absolutely sure: she gave [the HIVST] to me and she [the peer educator] said go and do it. She did not tell me to share.* Female, 29 years old, face-to-face interview, FSW, Côte d’Ivoire.

Finally, situations have occurred in which the number of HIVST kits allocated to the primary user for secondary distribution was inadequate, and in these cases, distribution to the main partner was privileged.

#### Primary user-level challenges to secondary HIVST distribution

Secondary distribution and the related challenges appear to differ across key populations as well as according to the relationship between the primary user and the potential secondary users.

#### HIVST distribution to main and regular partners

Among FSW, perceptions of partners’ sexual behaviours appeared to influence the prioritization of partners to whom HIVST kits are offered. FSW are more likely to offer the kits to partners with whom they do not use condoms and partners whom they assumed engaged in relationships with other women.

*When I saw my result [nonreactive], I took the second one [HIVST] there. I called my friend, who is a little bit frivolous too—he doesn’t stay quiet like me—I told him that he only has to do his test because he doesn’t like to use a condom. I know he does his own stupid things; I do my own stupid things, so I prefer to be protected.* Female, 32 years old, face-to-face interview, FSW, Côte d’Ivoire.

Among PWUD, the main reason that some participants did not distribute HIVST kits to their partners was because they were afraid of those partners’ reactions.

*Because I don’t know how she’s going to take it, I don’t know how she’s going to take it, so I never offered it to her*. Male, 35 years old, face-to-face interview, PWUD, Côte d’Ivoire.

Misinformation seemed to contribute to this fear. Indeed, according to one of the PWUD users who did not distribute an HIVST kit to his partner, only people who engage in risky behaviours should be tested for HIV, which would not be the case for his wife.

Among MSM, the anticipation of a possible negative reaction from the partner seemed to be a difficulty experienced by some. Two MSM who did not distribute kits to their partners motivated that decision based on their partners’ older age or because they did not identify as MSM.

*These are our partners who do not identify as MSM, so we are wary of giving the kits to these people because they might expose the secret [identity as an MSM] to the grins [a group of people who congregate to socialize, chat or play board games; the grin is often composed of people of the same sex and age group].* Male, 28 years old, face-to-face interview, MSM, Mali

Two MSM noted that they were concerned about the voluntary and confidential nature of HIVST. They opted to make a passive offer by talking to partners, displaying the kits, and waiting for them to express a desire to use them before offering them.

#### Secondary distribution to peers

MSM, FSW, and PWUD reported experiences with secondary distribution to peers (Table 3), with the numbers of kits distributed depending on the individual (8/21 MSM distributed it to peers, 7/24 FSW and 5/20 PWUD did it), the reactions of peers are uncertain, and/or participants might doubt that their peers will use the HIVST kits. Some interviewees with limited networks, such as PWUD living in ghettoes (reserved living spaces for drug users and other people involved with drugs), did less distribution of HIVST kits to their peers because they felt that since those peers were also present at the dispensing sites, they had already received them.

#### Secondary distribution to clients of FSW

FSW were less likely to offer the kits to clients with whom they did not experience fear of HIV infection given their consistent condom use. This rationale was expressed by a FSW, who said she never offered HIVST to her clients.

*In fact, I protect myself, and when the person protects himself, he is not at risk, unless the client breaks the condom*. Female, 32 years old, face-to-face interview, FSW, Senegal*.*

Condomless sex usually entails that clients pay a premium and FSW were more likely to offer HIVST kits to clients who request condomless sex. If clients have a reactive result, FSW can refuse to have sex without a condom. However, there are some barriers to distribution. For one FSW, the time that she has to spend with the client is too short to talk to him about HIV and testing; she also fears losing other clients if she spends sufficient time with each one to engage in HIVST distribution.

*But the client, to explain it to him, it will take an hour of time, and during that time I lose other clients; he too may have other activities to do.* Female, 32 years old, face-to-face interview, FSW, Senegal*.*

#### Secondary distribution to partners of users with reactive test results

Finally, secondary distribution appears to be difficult for individuals with reactive tests because of the issues of disclosing their HIV status. One FSW who determined her status through HIVST use reported sharing her result with her partner but failing to ensure that he was tested. One MSM with a reactive HIVST provided a kit to his partner but did not inform her of the nature of the test.

*Well, I didn’t tell her directly that it’s for AIDS testing, but I told her it’s a test for malaria and stuff, and she took it. So, when she got one line, I told her that she doesn’t have malaria, so that’s how I did it with her.* Male, 28 years old, face-to-face interview, MSM, Mali.

### Secondary users’ reactions to HIVST kit proposals

According to the experiences of primary users, the people to whom they offered the HIVST kits frequently accepted them. Some primary users reported that when they offered an HIVST to secondary users who were not familiar with or never head of HIVST, these secondary users were often, at start, surprised and curious, or reluctant and hesitant. But after explanation, they usually expressed enthusiasm for this new tool.

*Well, at first she doubted a little bit. She said, “But how can we be sure that her status can be known with this?”... I told her you’ll see. When you do, you will come and congratulate me. And then it was really the case. When she came, she thanked me.* Male, 44 years old, face-to-face interview, PWUD, Côte d’Ivoire.

This enthusiasm is justified as HIVST meets the needs of people who want to know their HIV status but are reluctant go to HIV testing facilities.

*There was one person who was sick, and it was not easy for him to get there. He was a bit old, and he said it had been a long time since he had been tested. So, I said “Wait, I have the HIVST for you”. I explained it to him, I played the video, I explained it to him, and he did it, so when he did it, he told me the result because he is a long-time friend.* Male, 35 years old, face-to-face interview, MSM, Mali.

Additionally, individuals with a fear of needles, especially in the MSM and PWUD communities, have found an alternative with the oral HIVST.

*The “branchés” [a term used in Côte d’Ivoire to refer to MSM]… they didn’t like the injection either. They didn’t like it at all. That’s why they had already stopped doing their tests for a while, even completely. Even if you come and say that you are going to prick them, they tell you “never”. So, when they saw the HIVST, they were really happy. They were really happy to do that.* Male, 21 years old, face-to-face interview, MSM, Côte d’Ivoire*.*

Trusting or influential relationships between partners are factors affecting the acceptance of HIVST through secondary distribution.

*He knows very well that I wanted him to do it. So, he asked me if this is what I really want, and I said yes, so he did it.* Male, 30 years old, face-to-face interview, MSM, Mali.

However, some refusals of HIVST kits were reported (five FSW with respect to their clients, two PWUD with respect to their peers, and two FSW with respect to their main partners). Fear of the testing result seemed to be the main reason for refusal.

When peers and partners refused to use the HIVST, it took the form of passive indifference.

*I told my husband about the HIVST. He asked me what the advantage of it is, and I told him that it is advantageous because when you do it and you see your result yourself, that is one of the advantages, but he never asked me to give him a kit.* Female, 20 years old, face-to-face interview, FSW, Mali.

With FSW’s clients, refusals could lead to an immediate break-up of the relationship initiated by either clients or FSW, as reported by three FSW. Verbal abuse by clients was reported by three FSW. Only one instance of physical abuse perpetrated by a client who had a reactive test was reported by a FSW.

“*They don’t want you [the FSW] to know their results. Maybe they are people who are already infected and they don’t want us to know*”. Female, 27 years old, face-to-face interview, FSW, Mali.

### Mechanisms for successful secondary distribution

In the aim of avoiding secondary users' negative reactions to offers of HIVST kits, some primary users developed strategies beforehand. The first strategy was to do an HIVST themself (and therefore knowing their own HIV status), before offering HIVST kits to others, so they could explain how to use it to others. Additionally, this allows them to know their current HIV status, and they could be tested again either “as a couple” or alongside the recipient without having doubts about their result, especially in the context of reluctant or hesitant partners or clients.

*My girlfriend told me that she prefers the evening, and that, when I come by, we’ll do it together. So, when I arrived, we did it together. She did not trust my test result, so I also did it again. I took the HIVST out, and we both did it.* Male, 39 years old, face-to-face interview, PWUD, Côte d’Ivoire*.*

Raising HIV/AIDS awareness, prior to initiating discussion of HIVST, is also a relatively common step among all three key populations.

*They [clients] would tell me that they are not sick, so I would tell them that nobody is sick. You just get tested to find out your status... But sometimes clients would get into conversations about AIDS with me, and I’d take the opportunity to tell them about the HIVST, until I could convince them to take it and do it at home.* Female, 35 years old, face-to-face interview, FSW, Senegal.

Finally, some key populations-specific techniques have been developed. For example, FSW often reported using certain methods to cajole their partners and clients.

*When I gave him the HIVST, he said that I don’t trust him...I joked with him, “Baby, that’s not it. You know, the life we lead here, really, it’s not that I don’t trust you, but you have to do it to see. That way, you are free, you at least know your status, you at least know that you are really healthy because first you have to know yourself, whether you are healthy or not. I spoke to him gently with soft, soft words*. Female, 24 years old, face-to-face interview, FSW, Côte d’Ivoire

With their clients, some FSW reported requiring HIVST as a condition for sex without condoms. Conditioning condomless sex on the partner performing the HIVST was also reported by some MSM, although not by PWUD.

*So, I told him, “If you don’t do [the HIVST test], we don’t have sex. Well, I convinced him, but he didn’t want to, so I came back home. That’s how he called me to tell me to come, that he was going to do [the test]. So, when he did the test, it came out negative.* Male, 24 years old, face-to-face, MSM, Côte d’Ivoire*.*

In situations in which communications about HIV with partners was difficult, passive distribution remained the preferred option for MSM.

### HIVST practices by secondary users

Data from the phone interviews enabled us to examine HIVST practices by secondary users who had received HIVST kits through the MSM or FSW distribution channels.

#### Delays between the receipt and use of HIVST

The majority of secondary users performed the HIVST within two days of receiving the kit, with some conducting it immediately upon receipt. Some of those waiting beyond that time period justified that delay by the innovative nature of the tool, which they did not fully trust. However, the most common reason for delaying the test was fear of the results, as noted by the following participant, who used his HIVST kit five days after receiving it.

*You don’t know what the result will be; it can make you hesitate... when he came back, he encouraged me and we did it... you know what this test is! No one, not even doctors, does this test without being afraid in advance.* Male, 43 years old, phone interview, MSM-based channel, Mali*.*

The longest delay observed was one month, as reported by a man who had received the HIVST kit from a colleague.

#### Empowerment in the use of HIVST kits

Approximately half of secondary users performed their test alone, without physical assistance (42%). Other secondary users required in-person assistance, which they usually sought from the primary user. Assistance could be requested for the purposes of psychological support or technical assistance.

From a technical point of view, requests for assistance were aimed at understanding the process of performing the test, assisting with the sample collection and/or interpreting the results. A few times, this assistance could either be total, with the primary user acting as a provider of HIV testing and conducting the whole process from beginning to the end, even announcing the result, or partial, as a means of meeting a specific need on the part of the secondary user. The latter was the case for the following participant, who required support from his partner with respect to interpretation of his HIVST test results.

*My concern was the interpretation of the lines. Then, he came to my house; he’s my cousin, we share the same life [they are both part of the MSM community] so we don’t hide anything from each other. I trust him completely.* Male, 30 years old, phone interview, MSM-based channel, Senegal*.*

The majority of secondary users who performed HIVST reported no particular difficulties (75%). The challenges mentioned by the other six participants were related to the process of collecting the buccal swab sample (2 participants) or interpreting the results (4 participants).

Question: And after you took the test, did you need to talk to anyone or get any specific information?

Answer: Well, when I finished taking the test, I needed an explanation of one part.

Question: What did you need to talk about?

Answer: It was part of the result display that I didn’t understand.

Male, 25 years old, phone interview, MSM-based channel, Mali, reactive test result.

With respect to the psychological aspect, the purpose of receiving assistance was to be provided with a reassuring presence. Indeed, few people sought support from the primary user simply because they needed encouragement to perform HIVST or because they had doubts regarding their ability to perform and interpret the results correctly. This was the case for one couple who received non-reactive results and the husband related the following during the telephone interview:

*I saw my result, I filed on the table, as I did not control that too much; I was not too happy I looked. I couldn’t laugh [rejoice], I couldn’t say a word; here I am. I thought, I’m going to call my guy [his friend who gave him the HIVST kits] so he can tell me if it’s good or not [reactive or not reactive]. As Mrs. [his wife] dropped off her kit next to me, when we saw the same things, she said here it is... here is the same line that is there. I said ok, let’s not rush, my guy will come and explain this.* Male, 45 years old, phone interview, FSW-based channel, Côte d’Ivoire*.*

Occasionally, fear of the test results motivated secondary users to request the primary user’s physical presence when performing HIVST. Such was the case for the following woman, who received her HIVST kit from her partner who had already used it.

Question: How easy or difficult do you think it is to do HIVST on your own?

*Answer: It’s easy.*

Question: What makes it easy?

Answer: When it’s explained to you properly, it’s easy.

Question: But why didn’t you do it by yourself after he explained it to you?

Answer: Because I was afraid.

Female, 25 years old, phone interview, FSW channel, Mali.

Support tools such as the video, the free hotline and the written materials (the information leaflet and supplementary brochure) were very useful for secondary users. Indeed, whether participants used HIVST alone or with the assistance of others, the majority referenced the support materials. Written materials were used more often than the hotlines and the video. According to participants, these written materials were those most often adopted by primary users during distribution. The video was rarely used. For two participants, the primary user’s explanations were sufficiently clear, and they did not need to use any tools. Two internet searches were conducted on the internet by two MSM-channel users to complement these explanations.

### Management of HIVST-reactive results and access to care services

Regardless of the result, users generally communicated it to the person who gave them the HIVST kit, whether or not they requested such communication. The majority of users expressed a feeling of accountability to the person who gave them HIVST kits, whether that individual was a peer educator or a primary user. This communication has the advantage of facilitating access to confirmation services in case of a reactive result.

*Since I had the phone number of the one who gave me the test, I told him that after doing the test, the two lines are red. He told me that means I have an infection, to go [to the health facilities] and take some medicine... I went, and they gave me some tablets.* Male, 24 years old, phone interview, MSM-based channel, Mali.

However, two secondary users who received a reactive result reported that they did not share their result with the primary user to maintain the confidentiality of their HIV status. One of these users used the hotline for referral, and the other went to a key population-friendly clinic.

Most frequently, the need to be informed of their “true” HIV status and to end their anxiety led members of key populations who had a reactive test result to seek confirmation within a short time period, either the same day or within three days of using the HIVST.

*Not more than three days [time taken before confirmation] because I couldn’t be quiet. I remembered it every moment.* Male, 28 years old, phone interview, MSM-based channel, Mali*.*

One person who received a test through the MSM-based channel reported waiting a month to confirm his results. Almost all participants who had a reactive test result had their result confirmed (7/8). Only one person reported not confirming his result because of his fear of having a blood sample drawn. He preferred to consider himself to be HIV-positive, irrespective of the confirmatory result.

*No. I haven’t left yet anyway. But there are others who went to get a needle and got the same result [confirmation of HIV positive], so I said to myself that the [HIVST] test doesn’t lie, everything they put on it is true... they went to get a needle and they came back with the same result. I don’t like to be pricked, so I can’t go.* Male, 36 years old, phone interview, MSM-based channel, Côte d’Ivoire.

This reasoning contrasted to that of one MSM who was interviewed face-to-face. He had never been tested for HIV due to a fear of needles. However, he was able to overcome his fear and use the confirmatory facilities when his HIVST result was reactive, and he received care.

All the individuals who confirmed their HIVST result had access to antiretroviral treatment.

## Discussion

This study documented the motivations, experiences, and challenges of MSM, FSW and PWUD who were provided HIVST kits for secondary distribution as well as the experiences of secondary users. We found that secondary distribution through key population can increase access to HIV testing for their partners, peers, and other relatives. Their motivation is to protect both others and themselves from HIV. Refusals to use HIVST was rare/not common and various reasons were provided for it. Secondary users often maintain contact with primary users and are able to complete HIVST with their help and/or the help of written materials, the video, or the hotline. Those with reactive results use confirmation and care services, with or without the help of those who provided them with the HIVST kit. The negative consequences of HIVST use are minimal.

Effective HIV secondary distribution to partners, peers, and other relatives was achieved by the majority of primary users. This result confirms the feasibility of secondary distribution by MSM, FSW, and PWUD [11]. The lower level of HIVST distribution to casual clients of FSW, who were merely “passing through”, compared to the level of distribution to regular non client partners was also noted in Uganda [7]. Because HIVST is a new tool, it is often met with a variety of reactions ranging from surprise to resistance, but typically, primary users have been able to convince secondary users to adopt HIVST. Key populations who have access to facilities or receive outreach services can be channels for reaching their partners, peers, and other relatives. Some countries are currently developing access to HIVST in private pharmacies for the general population. Once available, broad population-based communication on HIVST in the general population could be relevant, as it would also benefits to key population members. The need for such communication with the general population, even focusing on groups other than key populations, is justified by recent estimates that nearly 87% of the population in Africa is unaware of HIVST [30].

Secondary distribution is not always easy to perform and requires some degree of effort from primary users. They occasionally used clever strategies to convince others to use HIVST. In the case of regular partners, the classic couple testing strategy of first finding out one’s own status and then retesting together is often used by primary users who receive nonreactive results to overcome various kinds of resistance, or when such resistance is suspected [31]. This technique was common among all key populations: MSM, FSW, and PWUD. This suggests that, to encourage secondary distribution, at least two kits per key population member should be offered. Requiring HIVST completion as a condition for sex has been observed in the context of MSM partners as well as between FSW and their clients. This is different, however, from the coercion to complete HIVST reported by other studies. Bwaliya et al. reported that women pressured their husbands to test for HIV. In the same study, some women and young people experienced both pressure and coercion, with men or older people exerting their authority in the household to insist that their partner test for HIV using an HIVST kit. Some cases of coercion are also reported by Kumwenda et al. [32, 33].

Testing for HIV before sex to prove negative status, particularly when negotiating condom use, is potentially a risk compensation strategy. This practice is not completely safe, but in a context where negotiating a protected relationship is difficult it was the best one that some participants found to protect themselves. Even if some uses of HIVST are not "officially" recommended, people can adopt their own practices and do their own benefit/risk analysis. It is interesting to note that those who feel unable or unwilling to distribute HIVST kits contribute to the promotion of HIVST by raising awareness and providing referrals to HIVST supplies, thereby facilitating access to HIV testing. Fear of secondary users’ reactions was a common barrier faced by all three key populations but appeared to be more pronounced among PWUD concerning their life partners. Barriers to secondary distribution were most notable among PWUD and FSW, as no MSM mentioned a total refusal by a secondary user. Do MSM have other ways of selecting the members of their networks to whom they offer HIVST or more appropriate strategies for such proposals? Further studies are needed to explore this issue. As observed in other studies, responses to HIVST offers were generally positive [34]. Most studies have shown that the secondary distribution of HIVST does not result in severe negative consequences, a claim which was confirmed by our study [35, 36]. One FSW reported physical abuse.

Certain opportunities for HIVST distribution were missed. Some members of key populations did not have sufficient HIVST kits to ensure full secondary distribution to all members of their sexual networks; this scarcity was especially salient among FSW who have both partners and clients. In such situations, FSW prioritize their own sexual networks. While the emotions and the types of relationship with the secondary user (i.e., partner versus client) influence distribution decisions, the systematic use of condoms with the person in question is another. FSW prioritize the distribution of HIVST to people with whom condom use is inconsistent or absent. With FSW’s clients, the choice of “HIVST or condom” is pervasive and needs to be explored further. Indeed, the practice of testing clients who refuse condoms or are willing to pay a premium to engage in condomless sex appears to be adopted by some FSW. This practice has also been described elsewhere as a strategy to increase fees for sexual services [37]. The same practice seems to be used by some MSM and their partners. HIVST appears to be a new aspect of various risk reduction strategies. At this time, it remains unclear whether this strategy increases or reduces exposure to HIV. Condomless sex following a false negative result, or sex with a primary partner, might increase HIV-acquisition risk unless condomless sex would still have occurred in the absence of HIVST. In the latter case, HIVST would be a real risk reduction tool by preventing sex with individuals at risk of transmitting HIV.

HIVST utilization by secondary users is effective. The results show that some of the initial concerns raised by stakeholders in these three countries at the beginning of the project were unfounded [21]. Data taken from the phone interviews with participants who received the kits through the MSM and FSW channels demonstrate that the distributed kits are used. Although our study does not allow us to quantify the proportion of people who received an HIVST and did not use it, the literature on the subject suggests that they are few of them. In Uganda, 81% of FSW who were provided with HIVST kits reported using them [11]. In a pilot study in the same country, 95% of kits dispensed through secondary distribution were used by MSM [23]. Regarding the capacity of key populations to use HIVST without the support of “community or health experts”, some (6/24) secondary users in our study noted particular difficulties. In South Africa, a study confirmed the ability of MSM to perform oral tests correctly [9]. For the ATLAS project, the availability of support tools (video, written materials and toll-free telephone numbers), including materials in national languages may be partially responsible for this high capacity to perform the test. Contrary to the concerns that have been generally expressed regarding the illiteracy of populations in Africa as a limiting factor for the use of HIVST, the written and visual materials were consulted more frequently by users than were the video and the toll-free telephone numbers. In another study conducted in Senegal, more than 86% of users from (MSM and FSW) found the instructions to be sufficiently clear [13]. However, the ATLAS project supplemented this manual with a more explicit booklet explaining how to complete HIVST. In settings in which only written materials are available, secondary users could be able to complete their HIVST. However, these support tools alone may not meet the needs of users, as some secondary users needed a physical presence, not for the purpose of providing technical assistance but for moral support. This person could be a peer, just the primary user or phone trained operator. Therefore, it is relevant for programs to promote the possibility of physical support to HIVST secondary users when needed, through primary contacts redistributing kits or by giving the opportunity to call trained dispensing agents.

Reactive HIVST results are also confirmed. According to almost all interviewees with a reactive self-test, they have linked to confirmatory HIV testing (7/8), which often took place within a relatively brief period of time. Studies of secondary distribution among MSM have shown that they used care services [10, 23]. This result demonstrates that HIVST use encourages demand for care services when necessary. According to our study, the factors that encourage such demand were the need for members of key populations to know their serostatus, continued contact with primary users, and the availability of counselling through the anonymous hotline. Confirmation of a reactive result has led to antiretroviral treatment initiation.

### Strengths and limitations

Our results should be interpreted in light of the study’s limitations. First, the analyses were based on participants’ self-reports. Given the sensitivity of the topic (HIV and sexual behaviour), the nature of the respondents (populations who are at risk of HIV), and especially their double stigma of HIV and membership in a key population, some participants’ response could have been affected by social desirability bias. Few secondary users were interviewed, especially those from FSW channel and their statements in the telephone study may also have been influenced. Indeed, establishing a relationship of trust with a “stranger” over a short period of time is challenging. In the absence of such bond, it may have been difficult for participants to express themselves without reservations. Nevertheless, the preservation of anonymity may have encouraged disclosure. Additionally, not all participants received HIVST kits for secondary distribution or personal use at the same time, and the information collected is likely to change. For example, over time, some participants were able to overcome barriers to secondary HIVST distribution. Only three female PWUD were interviewed as primary users, and a gendered approach was not used for data analysis. Nonetheless, these analyses, which are among the first pertaining to secondary distribution of HIVST in West Africa, demonstrate its feasibility and acceptance by members of key populations in these countries. The results are specific to those key populations and their particular community-based setting. They could be different from health facilities where people are seeking care; health providers have less time to support them and incentivize their relatives to use HIVST.

Strengths of this study include use of standardized methodology across key populations and countries, as well as the consistency of our finding across settings, where there was little difference between participants’ point of view. Second, in settings of high HIV burden where key populations face considerable stigma, HIVST users including PLHIV accepted to share their experiences with a research team. Finally, it is notoriously difficult to survey secondary users. Through the broad range of research undertaken by the ATLAS project, including the current study, we were able to reach secondary users through the telephone contacts they left during the quantitative study.

## Conclusion

HIVST kits redistribution is common in MSM, FSW and PWUD communities. Secondary distribution is more likely to occur among sexual partners with whom condoms are not used and to peers of individuals belonging to key population. When such kits are not available, key populations are promoting HIVST and referring potential users to people or places where they can receive kits. The reactions of secondary users to HIVST proposals are generally positive, with very few adverse reactions (one physical abuse). People who receive HIVST via secondary distribution do the test relatively quickly after having it (2 days). In-person assistance is sometimes requested, for psychological support reason. These results support the deployment of HIVST to key populations, their partners and other relatives. In Central and West Africa countries with the same context, HIVST should be more widely and systematically accessible by FSWs, MSM and PWUD.

## Data Availability

The datasets for this manuscript are not publicly available because of conditions agreed with the participants, but are available from the corresponding author on reasonable request.

## Abbreviations

AIDS: Acquired Immunodeficiency Syndrome
COVID-19: Coronavirus disease
HIV: Human Immunodeficiency Virus
HIVST: HIV self-testing
MSM: Men who have Sex with Men
NGO: Non-Governmental Organization
PWUD: People Who Use Drugs
WCA: West and Central Africa
WHO: World Health Organization
